# Biomarkers and Pathogenesis of Infectious and Autoimmune Diseases

**DOI:** 10.3390/pathogens12010014

**Published:** 2022-12-22

**Authors:** Albert A. Rizvanov

**Affiliations:** Department of Genetics, Institute of Fundamental Medicine and Biology, Kazan Federal University, 420008 Kazan, Russia; rizvanov@gmail.com

Despite the fact that cardiovascular/ischemic diseases and cancers are major causes of death in the world, infections and autoimmune diseases also carry great burden to healthcare systems. The COVID-19 pandemic demonstrated that emerging and re-emerging infections put large populations at risk. In general, the immune response to an infectious agent is essential to fighting disease, restoring health and establishing long-term protection. However, abnormal activation of the immune response to pathogens, such as SARS-CoV-2, may lead to cytokine storm and damage to multiple tissues/organs, which became a hallmark of COVID-19, leading to severe disease or death. Moreover, in some cases, the immune response may lead to autoaggression, where leukocytes fail to properly process the antigen, leading to autoimmunity. Additionally, COVID-19 patients often suffer from complications, such as autoimmune complications, such as autoimmune hemolytic anemia, immune thrombocytopenic purpura, autoimmune thyroid diseases, Kawasaki disease, Guillain–Barre syndrome, etc. [1]. Moreover, little is known about the long-term effects of the autoantibodies detected after COVID-19 infection. Several manuscripts in the current Topical Collection address different aspects of COVID-19 immunity. Malisheni et al. studied mechanisms of neutralizing antibody evasion driven by non-epitope amino acid substitutions [2], while Bobik et al. describe antibodies in convalescent donor milk that may confer passive immunity against COVID-19 for infants [3].

Changes in cytokine levels are seen not only during COVID-19, but also for other viral infections. Martynova et al. demonstrated different patterns of serum cytokine, chemokine, and MMP levels in hantavirus-infected patients in different Volga regions of Russia, likely influenced by environmental factors and lifestyle differences, which affected individual immune responses to the infection [4].

The interplay between infections and the immune system is crucial not only for COVID-19 but also for many non-communicable diseases. Infections can be complications of many non-communal diseases. Nardone et al. describe a fatal case of a patient with non-Hodgkin´s lymphoma, where immune suppression led to cerebral HSV-1 vasculitis, causing progressive ischemic stroke [5]. This case report highlights the importance of the monitoring, prophylaxis and timely treatment of infections in immune-suppressed patients.

Not only can viruses cause life threatening infections, changes in the microbiome can serve as either a biomarker or cause of the disease. Balmasova et al. demonstrated specific microbiome patterns in patients with chronic periodontitis, chronic periodontitis associated with type 2 diabetes mellitus and healthy subjects [6]. Such changes in the microbiome might cause different immune responses in periodontitis patients. Bhalla et al. demonstrated elevated levels of complement effector molecule C5a in such patients, though its role on the pathogenesis of periodontal disease still needs to be investigated [7].

Shevelev et al. explored the role of antibiotic-resistant bacteria in the complications of treatment of purulent infections, burns, and trophic ulcers [8]. Antimicrobial and antifungal treatments were effective in controlling infections, inflammation, granulation, and epithelialization of wounds in vivo.

Borchsenius et al. reviewed the interplay between mycoplasmas and host cells [9]. Mycoplasmal infections cause changes in signaling pathways and might cause adverse immunological reactions and other pathological conditions.

One of the most important immune system organs which help to fight against bacterial infections is the spleen. Attina’ et al. reviewed the benefits of partial splenectomy over radical splenectomy, which is often used to treat various pediatric hematologic disorders, taking into account protecting patients from infections [10].

Mobilizing organisms to fight infections often relies on adjuvants to boost immune response. Ivanov et al. reviewed the effects of adjuvants on inflammasomes and their use in the prophylaxis and therapy of infectious diseases [11].

This is just a glimpse of the various topics covered by our Special Issue. We invite authors to continue to contribute original manuscripts, case reports, clinical trials and reviews focused on infectious disease and autoimmunity.

Potential topics include, but are not limited to:

- Recent discoveries in infectious pathogens (genetic, proteomic, etc.);
- In vitro and in vivo studies aiming to identify mechanisms of infectious diseases;
- Biomarkers for the diagnosis and therapy of infectious disease and autoimmune conditions;
- Transcriptome and proteome analysis of infectious pathogens and autoimmune conditions;
- Bioinformatics approaches to characterize infectious pathogens related to the clinical presentation, treatment efficacy and prognosis of various cancers;
- Novel therapeutic approaches for the treatment of infectious diseases and autoimmune conditions;
- Mechanisms of the immune system and evasion and development of autoimmunity.
